# Sharing Frailty-related information in perioperative care: an analysis from a temporal perspective

**DOI:** 10.1186/s12913-019-3890-y

**Published:** 2019-02-07

**Authors:** Daniel Fürstenau, Claudia Spies, Martin Gersch, Amyn Vogel, Rudolf Mörgeli, Akira-Sebastian Poncette, Ursula Müller-Werdan, Felix Balzer

**Affiliations:** 1 Department of Information Systems, Freie Universität Berlin, School of Business & Economics, Garystr. 21, 14195 Berlin, Germany; 2 Department of Anesthesiology and Operative Intensive Care Medicine, Campus Charité Mitte, Charité – Universitätsmedizin Berlin, corporate member of Freie Universität Berlin, Humboldt-Universität zu Berlin, and Berlin Institute of Health, Charitéplatz 1, 10117 Berlin, Germany; 3 Charité – Universitätsmedizin Berlin, Humboldt-Universität zu Berlin, and Berlin Institute of Health, Geriatric Research Group, Reinickendorfer Str. 61, 13347 Berlin, Germany; 4 Einstein Center Digital Future, Wilhelmstraße 67, 10117 Berlin, Germany

**Keywords:** Frailty, Perioperative care, Critical care, Intensive care medicine, Information technology, Temporal dynamics, Health data exchange, Geriatric medicine

## Abstract

**Background:**

Especially patients older than 65 years undergoing surgery are prone to develop frailty-related complications that may go far beyond the index hospitalization (e.g., cognitive impairment following postoperative delirium). However, aging-relevant information are currently not fully integrated into hospitals’ perioperative processes.

**Methods:**

We introduce a temporal perspective, which focuses on the social construction of time, to better understand existing barriers to the exchange of frailty-related data, targeting complexity research. Our chosen context is perioperative care provided by a tertiary hospital in Germany that has implemented a special track for patients over 65 years old undergoing elective surgery. The research followed a participatory modelling approach between domain and modelling experts with the goal of creating a feedback loop model of the relevant system relationships and dynamics.

**Results:**

The results of the study show how disparate temporal regimes, understood as frameworks for organizing actions in the light of time constraints, time pressure, and deadlines, across different clinical, ambulant, and geriatric care sectors create disincentives to cooperate in frailty-related data exchanges. Moreover, we find that shifting baselines, meaning continuous increases in cost and time pressure in individual sectors, may unintentionally reinforce – rather than discourage – disparate temporal regimes.

**Conclusions:**

Together, these results may (1) help to increase awareness of the importance of frailty-related data exchanges, and (2) impel efforts aiming to transform treatment processes to go beyond sectoral boundaries, taking into account the potential benefits for frail patients arising from integrated care processes using information technology.

## Background

The background section outlines the motivation and purpose of the study, reviews literature in the area of Information Technology (IT)-based inter-organization cooperation, and introduces our own theoretically informed perspective.

### Motivation and purpose

In Germany, approximately 16 million patients per year undergo surgical procedures, half of which are performed in patients over the age of 65 years [1]. These patients are especially prone to frailty – defined as a clinically recognizable state of increased vulnerability against stressors, often resulting from an aging-associated decline in reserve and function across multiple physiologic systems [2, 3]. Frailty has been recognized as an underestimated area of health care, affecting both the individual patient and society from a broad public health perspective [4, 5]. Based on a large nationwide sample of community-dwelling individuals aged 65–79 years in Germany, the prevalence of frailty and pre-frailty was found to be 2.3–2.8% and 36.9–40.4%, respectively [6].

However, aging-relevant information are currently not fully integrated into the perioperative process. Anesthesiologists, surgeons, and other health care professionals rely on comprehensive access to data, both from inside and outside the hospital, that reflect the patient’s individual status and risk with respect to frailty [7–10]. Even if these data are in parts electronically available, they are mostly scattered among multiple IT structures that are not semantically connected. While previous literature on IT-based interorganizational cooperation has noticed important issues related to conflicts of interests and power struggles of different stakeholders in the implementation and scaling of innovations [11–13], it says relatively little about underlying differences in temporal regimes and potential implications for stakeholders’ scopes of action. It has also failed to deliver a temporal explanation for sectoral differences among different health care stakeholders and the organizational and social complexities of frailty-related perioperative treatment.

The purpose of this study is to analyze the structure of a typical frailty-collaboration network from a perspective of temporal dynamics. Our research context is perioperative care provided by Charité - Universitätsmedizin Berlin, a German tertiary hospital in a metropolitan region, from now on referred to as “hospital”. This setting is particularly suitable because the Charité had recently implemented a special track for patients older than 65 years scheduled for elective surgery.

By focusing on frailty-related data exchanges and drawing on a participatory modeling approach, the study addresses typical challenges faced by healthcare implementation initiatives that are rooted in complexity [14]. It does so by mapping the structure of frailty-related data exchanges, illustrating the incentive structures, and underlying temporal regimes for different stakeholders within that process. We hypothesize that this can help to increase awareness of the importance of such data exchanges, and well as better inform efforts to transform treatment processes, so that they may strive to go beyond sectorial boundaries, in light of the potential benefits for frail patients arising from integrated care processes using information technology.

### Theoretical underpinnings

Our starting point is the work by Chiasson and Davidson [15], which emphasizes the value of information and organization research for the effective development, application, and use of IT to manage and coordinate health services. Health services that are of relevance in this context are inter-organizational in nature, support processes using data exchange, and usually associated with the introduction of new digital technologies and infrastructures. In the following, we will analyze studies in the field of IT-based inter-organizational cooperation and introduce further theoretical underpinnings.

### IT-based inter-organizational cooperation

To begin, we point out that interorganizational healthcare projects involve *multiple* stakeholder groups. In this respect, it becomes clear that the stakeholders often have different dispositions and expectations towards new technologies [16]. Some of the stakeholders will resist, as exemplified by Klöckner et al. [17] and Wessel et al. [18] for national e-health initiatives. Often, as exemplified by major delays in the German Electronic Health Card [18], it is the fate of promising initiatives that some stakeholders become reluctant to participate due to a potential loss of autonomy, while others, locked in a suboptimal position, wait longingly for their cooperation [19–22]. Therefore, it is necessary for the promotors and sponsors of such initiatives to carefully analyze, and potentially restructure, the incentives and relationships of the potential participants. As shown by nation-wide studies on healthcare information infrastructures in Scandinavian countries, continued stakeholder participation and flexibility in technologies and implementation strategies are prerequisites for successful adoption and use [13, 23–25].

There is work emphasizing that unfavorable factors, such as uneven distribution of *economic* costs and benefits [26, 27], could lead to social dilemmas, and abandonment of solutions that would have been globally advantageous [19]. Other works are concerned with the *sensemaking and shared meanings/goals* in the implementation of healthcare technology. Ideological conflicts among different stakeholder groups [28], a lack of collective sensemaking [23], and the absence a cohesive vision [22] hinder the implementation of novel technology. In a closely related issue, many authors emphasize problematic *power relations* between stakeholders, whereby hierarchical positions or networks allow some people to become more influential than others by providing access to valuable resources. This can in turn create resource dependencies [26], which have a strong influence on possible changes in roles, perceptions, and alliances [16]. Various works [12, 16, 27–30] indicate that power constellations often influence organizational change, shape the positions of stakeholders toward an initiative, as well as their willingness to participate.

Taken together, the current literature has highlighted barriers related to individual incentives and shared meanings/goals, as well as the rise of power struggles. The perspective of temporal dynamics and acceleration that we will present supplements these views, and explains individual and organizational incentives, as well as power struggles based on the temporal regimes that determine action.

### A temporal perspective on frailty-related information sharing

Within the healthcare system, the relevance of economic constraints has risen. At office visits in primary care, a very limited amount of time is dedicated to the doctor-patient relation [31], which also applies to the frail patients (cf. [32]). In Germany, this is motivated by a billing system that pays for number of patients treated, completely disregarding time spent speaking with the patient. Hospitals are another case in point. While the Diagnosis Related Groups (DRG) system has made lump sums per case the primary determinant of case coverage, short cycle times remain important, since it allows more patients to be treated in the same time [33]. In outpatient care, similarly, the nursing time is based on reference values (e.g., 5–10 min for facial care and shaving). Rehabilitation treatments are limited in time, and these periods are standardized (see for geriatric rehabilitation also [34]). Altogether, these examples show that time is an essential resource in the health care system.

Against the backdrop of these developments, it is unsurprising that time in the healthcare system is a resource that motivates inter-professional cooperation, but may also constitute a barrier to cooperation. Time pressure may be a symptom of deeper differences in organizational goals and time horizons [35], in the sense that it reflects individual evaluations of how to use time effectively, and how to prioritize time with respect to other possible activities. Therefore, we can deduce that time is not only in itself an essential barrier for interorganizational cooperation, but time may be an even more general institutional barrier, as stakeholders orient their decisions and actions to existing temporal regimes in their area of operation.

In this paper, we view time as not being fixed in nature but as a social construct [36], meaning that the perception of time is not an objective reality, but socially shaped and reshaped by the social environment. We argue that fundamental differences in temporal regimes of different stakeholders dealing with frailty in the healthcare system encourage dissociated patterns of activity, hindering progress. By temporal regimes, we refer to the organizing logic of activities in time. At first, this logic has a descriptive component, referring to the sequence, speed, synchronization, periodicity, and duration of activities [37], e.g. the fact that a frailty assessment takes *x* minutes. Secondly, this logic has a normative component, referring to the evaluation of different patterns of activity by individuals, which prioritize activities according to their perceptions of, and with respect to, multiple other possible activities [38], e.g. whether a caregiver finds it worthwhile to spend these *x* minutes on a frailty assessment, given multiple other tasks and obligations at hand. The interaction of both components constitutes a temporal regime, which enables a temporal coordination of distributed activities [37]. It is fundamental that this process creates predictability, for example, how long something takes, with which other tasks it is synchronized, and at what speed the task has to be performed. The temporal regime, as a framework for action, guides individuals’ actions in the form of individually perceived incentives and, at the same time, the individuals themselves react to and stabilize the temporal regime by their actions. This dynamic renders temporal regimes as socially constructed – in certain social groups and niches – and thus subject to power struggles.

Temporal regimes are frameworks for action, and as such can become stabilized and taken-for-granted. As suggested by Rosa [39], our age is characterized by a “shrinking of the present”, which means that the interlinked acceleration of technology, social change, and the pace of life initiate a self-reinforcing loop. Calling this the “acceleration cycle”, he argues that it paradoxically leads to a continuous decrease of the individual’s time, despite all the time that technological progress actually saves. The reason for this is shifting baselines, which means that the savings in time by technological progress is overcompensated by increasing expectations of other individuals, and the broader social system in which the individual is embedded. Individuals must keep up with the speed of other individuals having the same technological tools at their hands (think of email versus traditional mail). Therefore, in a certain community (e.g., all email users in organization X), a certain temporal regime can become established (e.g., response at the same day), developing its own dynamics over time while expectations continue to surge (e.g., anticipation of continuous availability).

Based on the foregoing observations, we suggest that conflicts of interest and power struggles in the healthcare system are often conflicts over temporal regimes. As argued by Wessel [31], power describes the ability to exert influence over resources. Thus, the fear of losing resources or the willingness to win new resources may be a powerful motivation for resistance. Time is an essential resource in the healthcare system, yet it is nothing objective, but rather shaped by individuals’ actions according to existing temporal regimes. Therefore, the change in temporal regimes associated with the introduction of coordinated care processes could trigger contests over resources. Altogether, it is therefore plausible to assume that power struggles are often motivated by conflicting temporal regimes and changes related to them.

Frailty is prime example because it requires a process in which care is integrated across several professions and structures of and beyond an institution, and thus especially interesting from the perspective of complexity research. Frailty is also interesting from a data sharing point of view, as it is a complex phenomenon that includes physiological, cognitive, and psychosocial components, together with standard data of clinical routine. The starting point is a social dilemma, describing that local stakeholders’ incentives are disconnected – to varying degrees – from global necessities, leading to a situation where data sharing does not take place. To overcome that dilemma, a better understanding of the structure of the collaboration network, temporal regimes, and of the stakeholders’ views on data-sharing are necessary. To that end, we employ an inductive case study approach as outlined by Yin [40].

## Methods

### Selection of research site

Within the scope of a transdisciplinary research project, perioperative care provided by Charité - Universitätsmedizin Berlin, a tertiary hospital in Germany was identified as a case study. The hospital was among the forerunners in integrating frailty in the treatment process, and was thus well suited to provide access to data and relevant knowledge. It is connected to both internal and external stakeholders involved in frailty data exchange, such as a geriatric hospital and various physicians in private practice, allowing for a piloting study of the entire system dynamics and relationships involved in frailty data exchanges.

### Participatory modelling

Our main data collection approach comprised meetings serving as an informal exchange of ideas and subsequent discussion including participatory modelling by the authors of this work, bringing together domain and modelling experts [41]. The goal of these meetings and workshops was to identify the relevant relationships and dynamics of the system, eventually resulting in a feedback loop model [42]. Domain experts included primarily medical doctors (*n* = 5) with expertise in anesthesiology (CS, RM, AP, UM, FB) as well as one scholar contributing from the perspective of health economics (MG). In the group of medical doctors, professional experience varied from residents to heads of departments. Since all domain experts were staff members of the hospital, aspects regarding external stakeholders that play an important role in this subject matter, as for instance referring physicians in private practice, etc., were based on the author’s personal experience dealing with those entities.

The modelling experts had a health-IT- and health economics background, and primarily contributed their knowledge about business dynamics and systems thinking, mainly from a managerial and business perspective [43]. During a period of 1 year (October 2016–September 2017), a total of 13 informal meetings were held at the hospital, whereby the early emphasis was on creating a mutual understanding of the problem. Later exchanges served to describe system relationships in more detail, including potential entrapments and future reorganization scenarios. These meetings were participatory in the sense that results were constantly shared between modelling and domain experts, and feedbacks were incorporated into new iterations of the models and scenarios. The team’s mutual knowledge base was also fostered by on-site visits, allowing the domain experts to reflect on and triangulate existing assessments [44].

### Feedback loop diagramming

As our prime analytical technique, we employed feedback loop diagramming [42]. In contrast to traditional system dynamics modeling, the application of the technique aimed primarily for a qualitative understanding of the system relationships and dynamics. In a managerial context, similar patterns of potential entrapments have been identified by Senge [45] and Repenning [46]. In combination with the participatory approach, the technique can be seen as a decision support method, which was intended to inform efforts toward process reengineering and organizational transformation.

## Results

This section first introduces the treatment process and stakeholder network, before discussing rationales for the current state, from the stakeholders’ perspective, of temporal regimes.

### Treatment process and stakeholder network

**Fig. 1:**
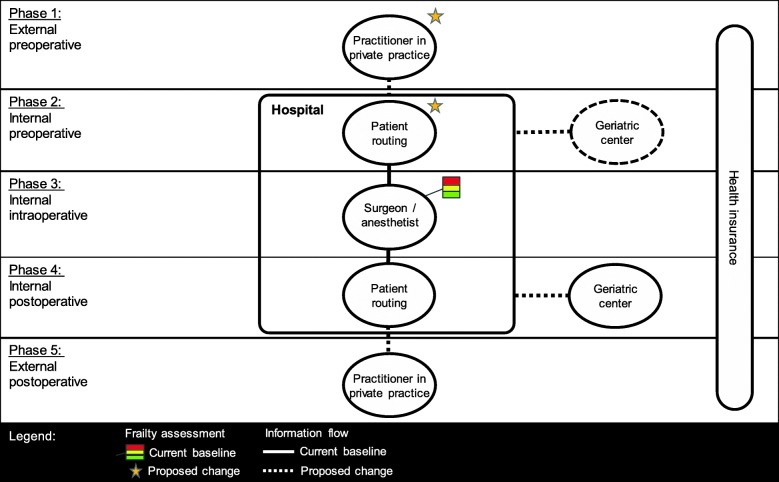
Stakeholder network for frailty-related surgical process

The case scenario describes the most common treatment process for patients undergoing surgical interventions, which are included in the special frailty track. Figure 1 shows a patient’s surgical process from consultation with a physician in a private practice, clinical assessment and work-up in the hospital, operation decision, operation, and subsequent postoperative treatment. In the figure, we depict the main stakeholders (as introduced before as part of the data collection strategy) and their interaction in a treatment process.

The depicted scenario deviates from the proposed target state, as defined in the participatory modelling workshops, in three essential points (see Table 1).

**Table 1:** Perceived benefits for sharing frailty-related information in perioperative care

Benefit / Stakeholder			1	2	3	4	Overview of perceived benefits
		…through identifying frailty-related complications		x	x	x	The identification of frail patients implies potential determination of related diseases and complications. The practitioners in private practice are primarily not affected by this benefit. Their role as the starting point in the process is to generate the data and detect further diseases in order for the subsequent stakeholders to adapt the patient treatment. The health insurance companies benefit through a more comprehensive information base on the insured patients.
Treatment quality and time	Treatment quality	… through implementing standardized decision processes		x	x		The alignment of the involved stakeholders with respect to the processes and database could enhance the treatment and decision-making processes. Standardization of the heterogenic database will cause high costs and effort for the practitioners in private practice. Being in the early stage of the process, they face the least potential benefit. From the current perspective, the healthcare providers are not concerned by the increase in data standardization, as it constitutes mainly a benefit for the operative stakeholders.
	Treatment time	… through avoiding repetitious procedures			x	x	Data sharing and the implementation of standardized data sets could reduce the number of medical tests. Repetitious procedures concern subsequent process steps and stakeholders, therefore not the practitioners in private practice and the geriatric center. The health insurance benefits financially from the elimination of repetitions.
		… through reducing waiting times for patients				x	The enhancement of the processes and data sets would ultimately have an impact on the waiting times between treatment steps. This mainly concerns the patient and partially the health insurance, as it might result in financial benefits.
Costs saving	Pre-operative	… through stratifying patients		x	x		Stratification enables the grouping of patients (e.g. high risk) and enables individual treatment processes. The geriatric center could be systematically integrated in the patient treatment, which is currently still not the case. Also, it increases process reliability for the surgeon / anesthesiologist, as they could base the treatment planning on differentiated information on the patients.
	Operative	… through avoiding operation cancellations			x		This leads to financial damages for the clinic (mainly surgery) and the health insurance. The early stratification of the patient enables the surgeons to consider a more comprehensive view on the patients and their resilience to overcome an operation.
	Post-op.	… through avoiding intensive care treatments			x	x	Intensive care treatments are related to uncertainty of the treatment planning, personnel expenditures (clinic) and high financial effort (health insurance). In relation to the stratification of patients, intensive care treatments could ultimately be avoided.

Perceived view from: 1…referring practitioners in private practice, 2...geriatricians, 3…surgeon/anesthesiologist, 4…insurance

Firstly, the initial frailty assessment should take place at an earlier time point. A timely identification of frailty would enable proper stratification, which in turn would allow the treatment processes to be adapted. Secondly, preoperative rehabilitative geriatric treatment should be established in order to improve patients’ conditions pre-operatively. In this way, treatment cancellation and postoperative complications are potentially avoided or reduced. Thirdly, the two aforementioned changes should be based on comprehensive data exchange. This data exchange would connect referring practitioners in private practice and geriatricians to the hospital, which could be the focal point of data exchange. Table 2 identifies a number of barriers to achieve the proposed process change, grouped by different stakeholders, which emerged through our participatory modelling workshops.

**Table 2:** Perceived barriers to sharing frailty-related information in perioperative care

Barrier / Stakeholder			1	2	3	4	Overview of perceived barriers
*Pressures of costs and efficiency*	*Time constraints*	Individual time constraints	x		x		On the operational level, the stakeholders face time constraints due to various reasons, which leads to a focus on the day-to-day activities, hindering systemic collaborations. Supposedly, this concerns mainly the referring practitioners in private practice and the surgeon/anesthetist at the hospital. It might be reasonable to take the geriatric center into account, but due to a lack of integration in the perioperative treatment, it is not concerned at this point.
	*Other resource constraints*	Lack of funding	x	x			Reimbursement concerns the re-payment for the conducted patient treatments. The geriatric center is not systematically included in the pre-operative patient treatment, raising reimbursement issues. Referring to the standardization of data sets and of processes, the alignment of the information systems and communication tools, it is apparent that data sharing requires a great amount of financial effort. Notably, the decentralized structure of ambulant sector (e.g. practitioners in private practice) requires early-stage financing to mount the innovation.
*Non-aligned/non-adapted action frames*	*Incompatible goals*	Incompatible organizational goals and time horizons	x	x	x		This refers to the divergent operational speed and the difficulties caused by these in the attempt of a cooperation. In this regard, this concerns primarily the stakeholders and cooperation between the practitioner in private practice, geriatric center, and hospital.
	*Legitimation and interpretation frames*	No legitimate action frame for preoperative treatment	(x)	x	x	(x)	The stakeholders face uncertainty regarding their action frame, due to a missing systemic perioperative process, the separation of the ambulant and clinical sector and the lack of financial support.
		Unclear interpretation of rules and responsibilities	x	x	(x)	x	Data security and the responsibility for the handling and exchanges of patient data remain unclear for the concerned stakeholders. Notably, by looking at the role of the practitioners in private practice in the treatment process (early stage, data generation and transfer) and their resources, it becomes apparent that this group of stakeholders faces the greatest challenges in understanding, interpreting and acting on the data protection guidelines.

Perceived view from: 1…referring practitioners in private practice, 2...geriatricians, 3…surgeon/anesthesiologist, 4…insurance

### Perceived individual barriers to data sharing

#### Referring physicians in private practice

Besides other issues, we perceived that referring practitioners in private practice may be reluctant to participate in data exchange due to *individual time constraints*, because the additional assessments would take time to prepare and execute. These time constraints, somewhat paradoxically, may lead to the fact that these stakeholders would prefer to save time, demanding solutions designed to make work easier and more efficient.

#### Hospital

The observations in the hospital brought individual time budgets to the fore, showing that time was not only scarce, but that time budgets were even exceeded. Doctors often work at their personal limit, and yet they were willing to do their best. Nonetheless, it appeared evident how useful it would be to save time and reallocate it between activities. The doctors especially strived for less time spent on administrative tasks, such as writing diagnoses in a patient file. This means that efficiency gains were expected, and frailty seemed promising in this extent. A commonly expressed goal was to reach a decision more quickly. At the same time, doctors at the hospital face a vicious circle, as this workload leads to time pressure, and is therefore perceived as a major barrier to cooperation. Put simply, the proposed change did not happen due to work overload.

#### Geriatrics center

The geriatric center, affiliated with the hospital and serving as a structure of early rehabilitation, worked in a different temporal regime. While time pressure was not as prominent as for the other stakeholders, it was a lack of funding and the lack of a defined preoperative treatment process, which let synchronization with the hospitals and referring practitioners in private practice appear elusive.

#### All stakeholder groups except insurances

For all stakeholder groups except for the insurances, divergent *organizational goals and time horizons* were regarded as major obstacle to cooperation. As the three stakeholders comprise an entirely different capacity of infrastructures and resources, the integration of these is perceived as barely realizable. It became clear that while working with another hospital was already challenging, it was even more difficult to cooperate across sectors, as these sectors are completely separate politically and in terms of health policy and administration.

Furthermore, these stakeholders operate within different temporal regimes and operational speeds. Undoubtedly, this also has an effect on their integrative capacity, as this implies a timely non-synchronized collaboration and creates difficulties at the interfaces. Especially in the case of emergencies, a coordination of different systems appeared difficult.

#### All stakeholder groups

A topic surrounding this problem of time has always been the *legitimate framework for action* and *unclear rules and responsibilities*. This concerned primarily medical evidence, without funding is not provided, but also data responsibility. There was substantial uncertainty on the side of the referring practitioners in private practice about the data transfer. Practitioners would be responsible for the accuracy of the data that they share. Probably additional measures of quality control need to be established, causing additional costs. It also creates additional responsibilities on the side of the hospital, in order enable data to leave the hospital.

## Discussion

The purpose of this paper was to analyze the structure of a typical frailty collaboration network in perioperative care from a temporal perspective. Frailty-related information sharing promises advantages in terms of treatment quality and time, as well as cost savings. There are, however, concerns related to operational pressures of costs and efficiency, most importantly time as a key constraint in the health care environment, and related to non-aligned / non-adapted action frames. Individual time constraints and other lacking resources create a disincentive, especially for practitioners in private practice, to invest in the information base that enables better decision-making. In addition, the non-alignment or non-adaptation of action frames and unclear rules and responsibilities operate at another level, because they represent institutional conditions, which once established as a framework for action have become themselves a source of rigidity, as the feedback loop model in the next section explains.

### Feedback loop model

**Fig. 2:**
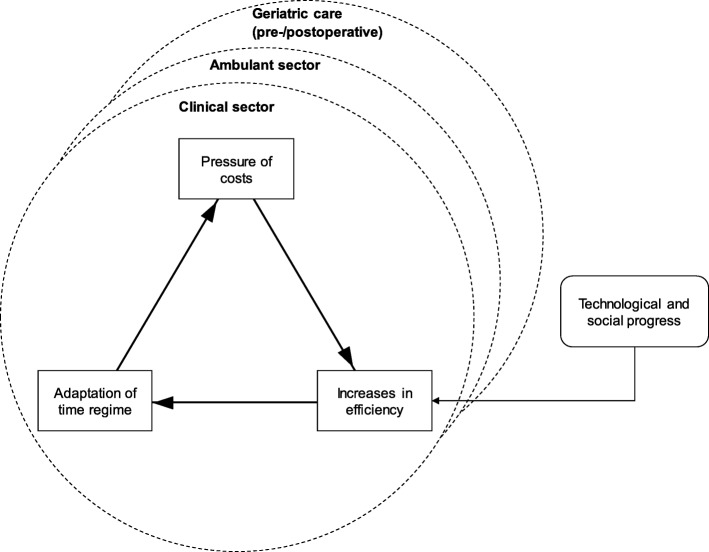
Dimensions of acceleration in the context of the perioperative treatment of frail patients

Turning to temporal regimes, the core of the model is the application of Rosa’s [39] analysis on temporality in modern societies (see section 2). Building on this view, we argue that the individual stakeholders in the German health care system, particularly hospitals (clinical sector), referring practitioners in private practice (ambulant sector), and geriatric care (pre−/postoperative) move in independent acceleration cycles, and have developed different temporal regimes. Nonetheless, the general structure of the acceleration cycles applies to all of them. As depicted in Fig. 2, they are in a treadmill of increasing cost pressure, the need to increase efficiency and in the course of time ever new adaptations, which affect their individual temporal regimes.

This may partially be explained by the structure of the German health care system. Hospitals are reimbursed through a lump sum per case (i.e., Germany has adopted a grouping system of patients based on the Diagnosis Related Group (DRG) system for hospitals; see [33]). As a result, hospitals are subject to a constant *pressure of cost*. Consequently, there is a focus on short-term performance improvement, which leads to an *increase in efficiency* by adapting the working methods and incorporating technological advancements. In consequence, the described “shrinking of the present” takes place: The efficiency-enhancing adjustments are carried out at both stakeholder (hospital) and environmental level (e.g., health insurance and governing bodies). This leads to an *adaptation of the temporal regime,* or shifting baselines, such as adjustments to case-related payments over time. These baselines, in turn, form the basis for individual action, in the sense that they create subjectively perceived incentives (e.g. for individual doctors in their daily routines). This action then stabilizes the temporal regime, because the healthcare providers tend to orient their actions towards the logic of cost savings and increased efficiency. As shown in the figure, this constitutes a self-reinforcing loop that leads to a continuous time compression dynamic, which generally affects all sectors but differs in its specific characteristics.

**Fig. 3:**
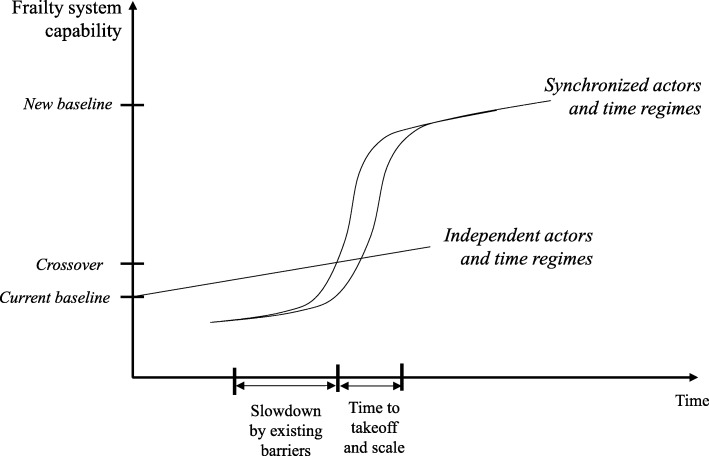
Capability of frailty system for different coordination modes and temporal regimes

Taking a closer look at the relationship between the practitioners in private practice and the hospital, it becomes apparent that these imply fundamental distinctions with regard to the individual time constraints and time horizons. The speed is very fast for hospitals. In the operating theater, every minute counts, which also applies to the billing of services. Referring practitioners in private practice are also inclined to be brief and to treat as many patients as possible, although for other reasons and motivations (see [47] for an ethical consideration of possible overtreatment in ambulant patients). These differences indicate that different, *independent* temporal regimes have been established. By that approach, and as indicated by the straight line in Fig. 3, only incremental gains in efficiency can be achieved.

The proposed process change, which was developed through a participatory modelling approach, might be associated with an improvement in treatment. However, this requires that the practitioners in private practice would become more actively involved in the role of a referrer and data supplier. Sectoral and professional boundaries would need to be at least partially resolved. The integration would require standardization to unify heterogeneous quality. Therefore, practitioners in private practice would transit to a situation of *dependence* and abandon some of their autonomy. The consequence would be an entrainment of temporal regimes, and possibly a different quality of acceleration in the ambulant sector. The same applies to geriatric clinics to a limited extent, although there is already a subtle synchronization through the postoperative process. The proposed process change, in the sense of a preoperative function, would require a stronger temporal coordination by means of harmonized systems and interfaces. In addition, the status of the geriatric center would change. The mentioned differences in organizational goals and time horizons indicate an increase in numbers of patients and an increase in administrative duties, which leads to an adjustment of the temporal regime. Overall, the dependence of the geriatric center and practitioners in private practice would increase. Both stakeholders would become “spokes”, while the clinic would play a central role in the temporal coordination of activities. This corroborates the assumption that through the centralization of processes and IT, the powerful position of the clinic would be strengthened, which, according to our data, could cause resistance among some stakeholders.

As shown in Fig. 3, the speed with which the implied target state can be achieved is described by the strength and intensity of the existing constraints in the form of time-based, institutional, and other barriers, such as the establishment of legitimate frameworks for action and the definition of responsibilities. The fundamental importance of these factors, for example, the unclear interpretation of data protection guidelines, is demonstrated by Wessel et al. [18] and is also evident in our analysis.

Based on a participatory modelling approach in one typical case setting, this analysis highlights the role of temporal regimes in the cooperation of different stakeholders in the healthcare system. Based on this perspective, we were able to show that independent temporal regimes create systemic hurdles that complicate extensive data exchange in the case of frailty. Our study was limited by the choice of methodology and by the selection of respondents. Further studies in the same fashion as ours should include interviews with stakeholders to validate the stated hypothesis, and expand the reach beyond a single site.

Several implications follow from our study. First, the finding presented may help involved stakeholders to redesign incentive structures. A main insight from the analysis is that measures that address only individual stakeholders in isolation are insufficient. This calls for systemic approaches that do not only target the dissolution of existing disincentives for individual stakeholders, but also encourage a broader discussion of existing conflict points.

The second implication concerns a better understanding of existing barriers and their partially self-reinforcing dynamic. Quite often in the literature, barriers have been understood as *spatial* boundaries, such as the height of financial and other differences between organizational sectors. However, it follows from our analysis that this view may be insufficient. Instead, we advocate for a *spatial-temporal* view, which acknowledges that different temporal regimes may exist, and that their dissolution requires changes to time structures. The temporal regimes are desynchronized in two independent sectors, and if these sectors are to grow together, synchronization of the temporal regimes are required. In order to synchronize them, the speed must be adjusted, i.e. one has to work faster or the other slower. Alternatively, the person who would have to work faster, in this case the practitioners in private practice, would have to be relieved of other tasks so that s/he could achieve more in the same time. Furthermore, it is possible that other professional groups, e.g. case manager, would help to create the necessary time to collect frailty data.

As a next step, we suggest the following future directions: first, a survey targeted toward practitioners in private practice, professionals from clinical and geriatric units, as well as patient groups, could be set up to investigate incentives on a larger population base; second, a simulation could contrast and compare the relative advantage of different scenarios taking into account path probabilities and costs. This study could be adjusted to find out how the rate of complications (or other relevant output measures, such as the length of hospital stays) must be decreased in order to justify the additional effort of perioperative treatment. Another fruitful path would be an analysis to determine how incentives must be redesigned in order to achieve an efficient solution for the involved stakeholders. This presents important research challenges – especially valuable for the growing number of frail patients.

## Conclusion

From a practical standpoint, frailty has extensive medical implications, which calls for integration of frailty assessment as early as possible in the perioperative process. This may only be feasible if incentive structures are realigned and benefits are clarified. Especially practitioners in private practices must be persuaded to participate in the data sharing and to create corresponding IT support. However, given the mentioned systemic hurdles (e.g., different temporal regimes), it may be more plausible to circumvent the problems by emulating the process entirely *within the hospital setting,* at least in the short and middle term.

## Abbreviations

DRG: Diagnosis Related Group
IT: Information technology

## References

[1] DESTATIS. Fallpauschalenbezogene Krankenhausstatistik (DRG-Statistik) Diagnosen, Prozeduren, Fallpauschalen und Case Mix der vollstationären Patientinnen und Patienten in Krankenhäusern [Internet]. Stat. Bundesamt, Fachserie 12 R. 6.4. 2014 [cited 2017 Mar 29]. Available from:https://www.destatis.de/DE/Publikationen/Thematisch/Gesundheit/Krankenhaeuser/FallpauschalenKrankenhaus2120640167004.pdf?__blob=publicationFile

[2] Xue Q-L. The Frailty syndrome: definition and natural history. Clin Geriatr Med. 2011;27(1):1–15. 10.1016/j.cger.2010.08.009. 10.1016/j.cger.2010.08.009 PMC3028599 21093718

[3] Fried LP, Tangen CM, Walston J, Newman AB, Hirsch C, Gottdiener J, Seeman T, Tracy R, Kop WJ, Burke G, McBurnie MA. Frailty in older adults: evidence for a phenotype. J Gerontol A Biol Sci Med Sci. 2001;56(3):M146-56. 10.1093/gerona/56.3.m146 11253156

[4] Buckinx F, Rolland Y, Reginster J-Y, Ricour C, Petermans J, Bruyère O. Burden of frailty in the elderly population: perspectives for a public health challenge. Arch Public Heal. 2015;73(1):1–7. 10.1186/s13690-015-0068-x. 10.1186/s13690-015-0068-x PMC4392630 25866625

[5] Morley JE, Vellas B, Abellan van Kan G, Anker SD, Bauer JM, Bernabei R, Cesari M, Chumlea WC, Doehner W, Evans J, Fried LP, Guralnik JM, Katz PR, Malmstrom TK, McCarter RJ, Gutierrez Robledo LM, Rockwood K, von Haehling S, Vandewoude MF, Walston J. Frailty consensus: a call to action. J Am Med Dir Assoc. 2013;14(6):392–97. 10.1016/j.jamda.2013.03.022. 10.1016/j.jamda.2013.03.022 PMC4084863 23764209

[6] Buttery AK, Busch MA, Gaertner B, Scheidt-Nave C, Fuchs J. Prevalence and correlates of frailty among older adults: findings from the German health interview and examination survey. BMC Geriatr. 2015;15:1–9. 10.1186/s12877-015-0022-3. 10.1186/s12877-015-0022-3 PMC4357063 25879568

[7] Amrock LG, Deiner S. The implication of frailty on preoperative risk assessment. Curr Opin Anesthesiol. 2014;27(3):330–35. 10.1097/ACO.0000000000000065. 10.1097/ACO.0000000000000065 PMC4076287 24566452

[8] Buigues C, Juarros-Folgado P, Fernández-Garrido J, Navarro-Martínez R, Cauli O. Frailty syndrome and pre-operative risk evaluation: A systematic review. Arch Gerontol Geriatr. 2015;61(3):309–21. 10.1016/j.archger.2015.08.002. 10.1016/j.archger.2015.08.002 26272286

[9] Lin H-S, Watts JN, Peel NM, Hubbard RE. Frailty and post-operative outcomes in older surgical patients: a systematic review. BMC Geriatr. 2016;16:157-169. 10.1186/s12877-016-0329-8. 10.1186/s12877-016-0329-8 PMC5007853 27580947

[10] Shem Tov L, Matot I. Frailty and anesthesia. Curr Opin Anesthesiol. 2017;30(3):409-17. 10.1097/ACO.0000000000000456. 10.1097/ACO.0000000000000456 28291129

[11] Currie WL, Guah MW. Conflicting institutional logics: a national programme for IT in the organisational field of healthcare. J Inf Technol. 2007;22(3):235–47.

[12] Constantinides P, Barrett M. Negotiating ICT development and use: the case of a telemedicine system in the healthcare region of Crete. Inf Organ. 2006;16(1):27–55. 10.1016/j.infoandorg.2005.07.001.

[13] Aanestad M, Jensen TB. Building nation-wide information infrastructures in healthcare through modular implementation strategies. J Strateg Inf Syst. 2011;20(2):161–76. 10.1016/j.jsis.2011.03.006.

[14] Plsek PE, Greenhalgh T. The challenge of complexity in health care. BMJ Br Med J. 2001;323:625–628. 10.1136/bmj.323.7313.625. 10.1136/bmj.323.7313.625 PMC1121189 11557716

[15] Chiasson M, Davidson E. Pushing the contextual envelope: developing and diffusing information systems theory for health information systems research. Inf Organ. 2004;14(3):155–88.

[16] Currie W, Pouloudi N, Whitley EA. Entangled Stakeholder Roles and Perceptions in Health Information Systems: A Longitudinal Study of the U.K. NHS N3 Network. J Assoc Inf Syst. 2016;17(2):107–61.

[17] Klöcker PN, Bernnat R, Veit DJ. Stakeholder behavior in national eHealth implementation programs. Heal Policy Technol. 2015;4(2):113–120. 10.1016/j.hlpt.2015.02.010.

[18] Wessel L, Gersch M, Harloff E. Talking past each other - a discursive approach to the formation of societal-level information pathologies in the context of the electronic health card in Germany. Bus Inf Syst Eng. 2017;59:23–40. 10.1007/s12599-016-0462-0.

[19] Ostrom E. Beyond markets and states: polycentric governance of complex economic systems. Am Econ Rev. 2010;100(3):641–72.

[20] Schröder S. Ökonomische Analyse und Bewertung integrierter Versorgungssysteme im Gesundheitswesen - Ansätze einer methodischen Erweiterung aus diffusionstheoretischer Perspektive. Dissertation. Berlin: Freie Universität Berlin; 2015. Available from: https://refubium.fu-berlin.de/handle/fub188/7896.

[21] Furstenau D, Wessel L, Gersch M. How organizational path constitution prepares digital infrastructure innovation: a case study of integrated care. Proceedings of the 22nd Americas Conference on Information Systems, AMCIS 2016, San Diego, CA, USA, August 11-14, 2016.

[22] Wessel L, Gersch M. From ICT to integrated care: the performative cohesion of organizing visions. Proceedings of the 23rd European Conference on Information Systems, ECIS 2015, Münster, Germany, May 26-29, 2015.

[23] Aanestad M, Jensen TB. Collective mindfulness in post-implementation IS adaptation processes. Inf Organ. 2016;26(1-2):13–27. 10.1016/j.infoandorg.2016.02.001.

[24] Aanestad M, Grisot M, Hanseth O, Vassilakopoulou P. Information infrastructures for ehealth. Information infrastructures within European health care: working with the installed base. Cham: Springer Open; 2017. 10.1007/978-3-319-51020-0_2. 31314222

[25] Hanseth O, Bygstad B. Flexible generification: ICT standardization strategies and service innovation in health care. Eur J Inf Syst. 2015;24(6):645–63. 10.1057/ejis.2015.1.

[26] Pfeffer J (1981). Power in organizations.

[27] Palvia P, Lowe K, Nemati H, Jacks T. Information technology issues in healthcare: hospital CEO and CIO perspectives. Commun Assoc Inf Syst. 2012;30:293–312. 10.17705/1CAIS.03019.

[28] Paul RJ, Ezz I, Kuljis J. Healthcare information systems: a patient-user perspective. Heal Syst. 2012;1(2):85–95.

[29] Boonstra A, Boddy D, Ball S. Stakeholder management in IOS projects: analysis of an attempt to implement an electronic patient file. Eur J Inf Syst. 2008;17(2):100–11.

[30] Cavazza M, Jomni C. Stakeholders involvement by HTA organizations: why is so different? Health Policy (New York). 2012;105(2-3):236–45. 10.1016/j.healthpol.2012.01.012. 10.1016/j.healthpol.2012.01.012 22364715

[31] Tai-Seale M, McGuire TG, Zhang W. Time allocation in primary care office visits. Health Serv Res. 2007;42(5):1871–94. 10.1111/j.1475-6773.2006.00689.x. 10.1111/j.1475-6773.2006.00689.x PMC2254573 17850524

[32] Tai-Seale M, McGuire T, Colenda C, Rosen D, Cook MA. Two-minute mental health care for elderly patients: inside primary care visits. J Am Geriatr Soc. 2007;55(12):1903–11. 10.1111/j.1532-5415.2007.01467.x. 10.1111/j.1532-5415.2007.01467.x 18081668

[33] Schreyögg J, Stargardt T, Tiemann O, Busse R. Methods to determine reimbursement rates for diagnosis related groups (DRG): A comparison of nine European countries. Health Care Manag Sci. 2006;9(3):215–23. 10.1007/s10729-006-9040-1 17016927

[34] Busetto L, Kiselev J, Luijkx KG, Steinhagen-Thiessen E, Vrijhoef HJM. Implementation of integrated geriatric care at a German hospital: a case study to understand when and why beneficial outcomes can be achieved. BMC Health Serv. Res. 2017;17(1):180-94. 10.1186/s12913-017-2105-7. 10.1186/s12913-017-2105-7 PMC5341181 28270122

[35] Boothroyd RA, Evans ME, Chen H-J, Boustead R, Blanch AK. An exploratory study of conflict and its management in systems of care for children with mental, emotional, or behavioral problems and their families. J Behav Health Serv Res. 2015;42(3):310–23. 10.1007/s11414-014-9448-1. 10.1007/s11414-014-9448-1 25391358

[36] Bergson H. Time and free will: an essay on the immediate data of consciousness. North Chelmsford: Courier Corporation; 2001.

[37] Thompson EP. Time, work-discipline, and industrial capitalism. Past & Present. 1967;38(1):56–97.

[38] Luhmann N. The future cannot begin: temporal structures in modern society. Soc Res. 1976;43(1):130–52.

[39] Rosa H (2013). Social acceleration: A new theory of modernity.

[40] Yin RK. Case study research: design and methods. 5th ed. Thousand Oaks: Sage Publications; 2013.

[41] Basco-Carrera L, Warren A, van Beek E, Jonoski A, Giardino A. Collaborative modelling or participatory modelling? A framework for water resources management. Environ Model Softw. 2017;91(1-3):95–110. 10.1016/j.envsoft.2017.01.014.

[42] Perlow LA. The time famine: toward a sociology of work time. Adm Sci Q. 1999;44(1):57–81.

[43] Sterman JD (2000). Business dynamics: systems thinking and modeling for a complex world.

[44] Locke K, Golden-Biddle K, Feldman MS. Perspective--making doubt generative: rethinking the role of doubt in the research process. Organ Sci. 2008;19(6):907–18. 10.1287/orsc.1080.0398.

[45] Senge PM. The fifth discipline: The art & practice of the learning organization. New York: Broadway Business; 1990.

[46] Repenning NP, Sterman JD. Capability traps and self-confirming attribution errors in the dynamics of process improvement. Adm Sci Q. 2002;47(1):265–95. 10.2307/2667031.

[47] Strech D. Der Abbau von Überversorgung als Teil der ärztlichen Berufsethik. Z Gerontol Geriatr. 2014;47(1):17–22. 10.1007/s00391-013-0590-9. 10.1007/s00391-013-0590-9 24389719

